# Department of Surgery

**Published:** 1901-05

**Authors:** W. E. A. Wyman, J. A. Sloan, Robert Jay

**Affiliations:** Milwaukee, Wis.; San Francisco, Cal.; West Liberty, Ia.


### DEPARTMENT OF SURGERY.

By W. E. A. Wyman, M.D.V., V.S.,
MILWAUKEE, WIS.

J. A. Sloan, B.Sc., M.D.V.,

SAN FRANCISCO, CAL.

Robert Jay, M.D.V.,

WEST LIBERTY, IA.

### Hoof Diseases.

(Continued from page 239.)

### Pododermatitis.

The prognosis of serous pododermatitis is based upon numerous factors. Those animals having faulty hoofs or a malposition of the limbs are predisposed to the disease, and can at best be temporarily relieved, while serous pododermatitis following mechanical injuries is, as a rule, curable. The prognosis, therefore, in such cases is favorable. Of course, when the disease is of old standing and distinct anatomical changes have occurred little can be done; while excessive pulsation and decided œdema, which at the same time mean great pain and thus lameness, reasonably call for a guarded if not an unfavorable prognosis. It is also to be remembered that serous pododermatitis of the fleshy sole and frog yield more readily to treatment than that of the coronary cushion. In cases of a chronic kind, where marked anatomical changes have taken place, no cure is to be expected ; such animals can only be made serviceable.

Remove its cause and its effects cease is naturally the *conditio sine qua non* in the therapeutics of serous pododermatitis. Whenever the animal is predisposed to the disease, by reason of inferior hoofs or of malposition of its limbs, removal of the cause is practically impossible, and all means employed can necessarily give but temporary relief. The essential feature in the treatment of pododermatitis is clearly rest ; the extent and the intensity of the disease increases with exercise, since it irritates the parts again and again.

In the very earliest stages of the disease the application of cold is indicated on account of its contracting effect upon the blood-vessels and its effect upon the immigration of the white blood-corpuscles. In the hoof cold only exerts a material influence upon the region of the coronary cushion and frog. According to Bayer's experiments constant irritation is best. By means of a very ingenious contrivance Bayer took the temperature of a hoof at

101.3° F., and irrigated it with water of a temperature of 43.7° F. In five minutes the inner surface of the horny box which held the thermometer showed a temperature of 100.7° F.; in ten minutes a temperature of 98.6° F., and after one-half hour's irrigation it registered 93 2° F. This plainly shows the limited effect which even such cold water has upon lowering the temperature of the hoof, especially when it is taken into consideration that the experiment was carried on with a dead hoof, while in the actual living hoof the effects of even a continuous application of cold water must be less, as the blood would absorb a great deal of the cold and carry it away through its circulation. Of the many appliances which are at our disposition for constant irrigation in the treatment of these cases, a cloth wrapped around the hoof with a hose pushed under it and connected with the hydrant is as good as any complicated or costly arrangement. In case no flowing water can be had the less effective cold-water swabs may be employed, made either of felt or a piece of some old street blanket, but in this case the material should be moistened not simply with cold water, but with some disinfectant solution, as the presence of more or less small cracks about the hoof might lead to an infection and to dangerous complications. Another admirable way to apply cold is the ice-pack. Small pieces of ice are mixed with sawdust, the horse's hoof and foot placed either into a regular boot or a trouser leg, and the chopped ice packed around the parts. In the country a flowing brook answers the purpose nicely, while in the larger barns of cities a regular irrigation stand with cement bottom is used. As previously stated, the application of cold is only indicated in the earliest stages of the disease.

After the trouble has existed for several days no good can follow the use of cold, and the application of heat becomes essential. Moist heat is used in the shape of poultices of linseed meal, which should always be made up with some disinfectant for the reasons just mentioned; about one pound per hoof is sufficient. These are to be continued for about one week. Before the cataplasm is applied it is absolutely necessary to grease the hollow of the heels, the bulbs, and the coronet with some bland lardaceous material. By so doing one prevents too intense a maceration of the epidermis and horn, which render them so unyielding that they give way to the increased transcapular pressure caused by the inflammatory process, and in consequence bulge out and, as already stated, lay the foundation for a coronary contraction. The writer has used for years with most gratifying results cotton-waste moistened with rather hot water containing some antiseptic instead of the regulation linseed poultices; a thick layer of the cotton-waste is placed under and around the hoof properly secured and moistened by simply pouring the hot water upon it every three or four hours. It represents a much clearer and decidedly more surgical means than all other poultices so far tried. Among certain people belonging to the genus "sus" poultices made of cow and other manure are still used. The impropriety of these germ-laden, nasty applications is too apparent.

As soon as the cataplasms can be dispensed with the whole hoof, sole, frog, and all should be dressed with some oleaginous preparation to prevent the too rapid evaporation of the water artificially introduced by the poultices, and this followed by a light blister to the coronet. On the whole, it is not necessary to practice phlebotomy, but should the full bounding pulse and other indications make the abstraction of blood advisable it is best to open the jugular vein and not to bleed from the toe. This has sometimes given the writer most excellent results. Nevertheless, the chances of a secondary infection are so many that unless an aseptic operation with proper subsequent precautions is possible the method of abstracting blood is best left alone.

As regards the removal of the shoe in the treatment of serous pododermatitis no absolute rule can be established. As the wall of the horse shod regularly is generally cut down pretty well the animal is likely to have better footing with the shoe on than with it removed, while it would be positively wrong to remove the shoe in the long-toed, acute-angled, weak-heeled, flat-soled, or drop-soled hoof.

In regard to internal medication but little need be done, though an aloes pill sufficiently large to act as a laxative, but a laxative only, is indicated. Of course, in many cases the owner of such an animal is not at all satisfied unless he can give the creature some medicine regularly, which emergency the practitioner must meet according to his judgment.

(To be continued.)

---

Messrs. P. Blakiston's Son & Co. have just issued a little book, entitled *Self-examination*, for medical students, containing 3500 questions on medical subjects, intended as a self quiz-master. The book is gotten up in a compact form and should be of great assistance to those contemplating final examinations. It is published at the nominal sum of ten cents.

